# Feasibility of Linking Molecular Genetic Markers to Real-World Social Network Size Tracked on Smartphones

**DOI:** 10.3389/fnins.2018.00945

**Published:** 2018-12-18

**Authors:** Rayna Sariyska, Eva-Maria Rathner, Harald Baumeister, Christian Montag

**Affiliations:** ^1^Institute of Psychology and Education, Ulm University, Ulm, Germany; ^2^MOE Key Lab for Neuroinformation, The Clinical Hospital of Chengdu Brain Science Institute, University of Electronic Science and Technology of China, Chengdu, China

**Keywords:** *Personality Neuroscience*, molecular genetics, oxytocin, oxytocin genetics, extraversion, smartphones, *Psychoinformatics*, digital phenotyping

## Abstract

The study of individual differences in human social behavior has a long tradition in (personality) psychology focusing on traits such as extraversion linked to vividness and assertiveness. The study of molecular genetic underpinnings of individual differences in social behavior produced many genetic association studies with only few genetic variants, robustly associated with individual differences in personality. One possible reason for non-replication of findings might be the different inventories used to assess human social traits. Moreover, self-report methods to assess personality and social behavior might be problematic due to their susceptibility to different biases such as social desirability or poor abilities in self-reflection. We stress the importance of including recorded behavior to understand the molecular genetic basis of individual differences in personality and linked social traits. We present preliminary data linking oxytocin genetics to individual differences in social network size derived from smartphones. Here, the genetic variation rs2268498, located in the adjacent area of the promoter of the gene coding for the oxytocin receptor (OXTR), was linked to the number of active contacts and incoming calls, tracked on the smartphone for 12 days (note that these results became a bit weaker when age was controlled for). Although the present empirical findings should only be seen as a proof of concept study, this work demonstrates the feasibility to combine molecular genetic variables with real world behavior. If this approach keeps its promises, the field of personality research might experience a boost in psychometric quality in the near future.

## Introduction

Disentangling individual differences in personality and intelligence represents an old quest, going back to the days of Sir Francis Galton, who was an early advocate of the use of twin studies (Montag and Hahn, 2018). Currently, abundant research is available demonstrating that individual differences in the mentioned areas are shaped by both nature and nurture. Per rule of thumb about 0.50 on the genetic and 0.50 on the environmental side impact individual differences in human traits as carved out in a large study reviewing 2,748 twin studies published between 1958 and 2012 (Polderman et al., 2015).

A logical next step from this branch of research would be to estimate the heritability of individual differences in a given trait such as personality through the localization of distinct areas on the human genome linked to individual differences in traits such as extraversion or neuroticism. This kind of research has started over 20 years ago being either pursued via the candidate gene approach or genome wide scan association studies (for an overview see Montag and Reuter, 2014). Until today the study of the molecular genetic basis of personality struggles with many problems, perhaps the greatest struggle is to still see only few genetic variants to be robustly associated with personality traits (for a recent overview on genome wide scan studies see Sanchez-Roige et al., 2018). One of the problems clearly has been underpowered, small sample-size studies (see for an overview also Munafò and Flint, 2011). Therefore, a recent attempt is noteworthy, that came up with reproducible gene-personality associations, but needed to include more than 329,000 participants from a United Kingdom biobank to observe 15 SNPs being robustly linked to neuroticism (Luciano et al., 2018). Of note, from our perspective this does not mean that the candidate gene approach is not able to produce robust associations (although others see this differently, e.g., Jern et al., 2017). For example, a highly cited meta-analysis observed that the interaction between the prominent 5-HTTLPR polymorphism and adverse environmental effects on negative emotionality seems to be stable (Karg et al., 2011). Recent work presented a new promising research paradigm in the context of the candidate gene approach, namely linking genetic variations to individual differences in personality in independent samples stemming from different ethnic groups, probably hinting at globally valid effects (Montag et al., 2017b; Sindermann et al., 2018).

In sum, different routes to the study of the molecular genetic basis of personality might ultimately be successful, but without doubt the “hunt” for genetic variants underlying personality is still challenging. This clearly is also due to (a) the polygenetic nature of personality, potentially influenced by several hundreds or even thousands of genetic variations all with small to tiny effects (see also recent advances stressed by Plomin and von Stumm, 2018) and (b) the type of personality assessment used in a respective study. Moreover, often not the same inventories and/or personality assessments are applied, making the comparison of results across genetic association studies even more problematic. In addition, most of the studies assess traits “only” via self-report, ergo problems such as social desirable answers (Van de Mortel, 2008) or not being able to remember previous events correctly (Stone and Shiffman, 2002; Montag et al., 2015a) might bias the data. While the polygenetic nature of personality needs consistent research efforts on a large scale, the limited psychometric quality on which personality research is often based, jeopardizes a whole research area.

Therefore, we aim to present in this short communication *preliminary data* on a new way to assess personality and, thus, to conduct research in the field of *Personality Neuroscience*. We already stress at this point that the presented empirical data of this work should be seen as preliminary, because the sample size is not sufficient to produce a stable outcome. On the other hand collecting the present data took more than one and half years with the recruiting of more than 100 participants providing us with insights into their objectively measured smartphone behavior and molecular genetic variables. Therefore, the present work should be understood as a study testing the feasibility to combine molecular genetic information with real-world behavior, tracked on smartphones, giving insights into individual differences in extraversion-linked smartphone variables.

In earlier works it was demonstrated that in particular call variables (Montag et al., 2014; Stachl et al., 2017), the use of social messengers such as WhatsApp (Montag et al., 2015b), but probably also the here investigated size of a person’s network are linked to extraversion (see the importance to assess age in this context, Roberts et al., 2008). The latter assumption is based on existing literature, reporting a link between high extraversion and the number of “friends” and memberships in different groups on Facebook (Ross et al., 2009; Amichai-Hamburger and Vinitzky, 2010). Extraversion itself is a personality trait closely linked to gregariousness, but also assertiveness, to name a few (Costa and McCrae, 1992). As the size of the social network of a person might be linked to the oxytocinergic system (Pearce et al., 2017; for problems with this work see Jern et al., 2017), the present study focused on the investigation of a polymorphism on the oxytocin receptor (OXTR) gene and individual differences in social network size. For the present work we hypothesized that the prosocial TT variant of the OXTR gene, linked to lower autistic traits in both Germany and China (Montag et al., 2017b), higher empathy (Christ et al., 2016), higher abilities in face recognition (Melchers et al., 2013) and processing of social information (Melchers et al., 2015), would be linked to having also a higher number of (active) telephone contacts in the smartphone and more active call behavior.

## Materials and Methods

### Participants

Smartphone and genetic data was available from *N* = 117 participants (77 females), mostly with a student background. The average age of participants was 23.04 years (*SD* = 7.32) and 76.9% reported having A level as their highest educational qualification. Most of the participants were recruited at Ulm University and signed an informed consent prior to participation in the study. They received university credits or monetary compensation for their participation. The study was approved by the local ethics committee of Ulm University, Ulm, Germany.

### Materials

The application *Insights* (an Android-based smartphone application, developed by Christopher Kannen^1^) was installed on participants’ phones either by the examiner or the participants themselves. This application records different variables such as the number of calls per day (incoming, outgoing, missed), the use of different applications such as YouTube or how active a person is (distance per day measured using the GPS function on the phone) etc. In the current study the number of contacts (names saved in the phone book, as well as the total phone numbers) and the average number of contacts one is in touch with per day through calls/actively used contacts per day (referred to as “active contacts”^2^) were used to measure the size of the social network. Furthermore, the average number and duration of calls per day (including the incoming, outgoing and missed calls) were considered as an additional measure. Twelve days of recordings were used to build an average of the tracked variables.

The genotyping was conducted at Ulm University. DNA was extracted from cell material via buccal swabs. DNA purification was conducted by means of the MagNa Pure 96 system (Roche Diagnostics) and genotyping via a Light Cycler Cobas z480 (Roche Diagnostics, real-time quantitative PCR and subsequently high resolution melting; Primer Assays by TibMolBiol) and a mass spectrometer MassARRAY (Agena Bioscience / Sequenom). Participants were genotyped for rs2268498, a functional single nucleotide polymorphism (SNP) on the OXTR gene (Reuter et al., 2017), positioned on chromosome 3p25.

Participants filled in a short version of the Trait-Self Description Inventory (TSDI, for the German version see Olaru et al., 2015), consisting of 42 items rated on a seven-point Likert scale (1 = strongly disagree to 7 = strongly agree). Only the personality characteristic extraversion was used in the analyses of the present study. Cronbach’s Alpha was α = 0.77.

### Statistical Analyses

The distribution of the variables was examined by assessing the skewness and kurtosis of the variables (Miles and Shevlin, 2001). Since all smartphone variables and age deviated from the normal distribution, non-parametric tests were applied. The CC and CT genotypes of rs2268498 were combined to a C+ group and compared to the TT genotype (C- group), according to our hypothesis. Since both age and gender have been linked to recorded smartphone use (see studies by Montag et al., 2014; Stachl et al., 2017), these associations were also tested in the present study. Spearman’s correlations were used to examine the link between age and the investigated smartphone variables. Mann–Whitney *U* test was applied to compare male and female participants with respect to the investigated smartphone variables and to assess the association between the rs2268498 genotypes and the smartphone variables. Where there was a need to control for age, the respective dependent variables were normalized using Blom rank-based transformation (Solomon and Sawilowsky, 2009) and an ANCOVA was conducted.

## Results

The distribution of rs2268498 genotypes did not deviate from the Hardy-Weinberg equilibrium (*X*^2^ = 0.22, *p* = 0.64). *N* = 27 participants were CC-carriers, *n* = 61 were carriers of the CT-genotype and *n* = 29 of the TT-genotype. According to our grouping 29 participants (TT or C-) were tested against *n* = 88 C+ carriers (CC + CT).

In Table 1 the descriptive statistics of the investigated variables are presented (including the median due to the non-normal distribution of the variables).

**Table 1:** Descriptive statistics for the investigated variables.

	Total contact names	Total phone numbers	Active contacts mean	Calls count	Incoming calls	Outgoing calls	Missed calls	Call duration min.
Mean	205.06	219.23	1.15	2.15	0.45	1.33	0.36	7.50
Median	180.00	190.00	0.83	1.33	0.25	0.67	0.25	4.10
SD	115.05	124.47	1.13	2.38	0.52	1.72	0.36	9.86
Min.	20	21	0.00	0.00	0.00	0.00	0.00	0.00
Max.	700	773	8.33	15.50	3.58	10.67	1.67	51.24


*N = 117, SD = standard deviation; Min. = minimum; Max. = maximum.*

The correlation analysis demonstrated that the variables *active contacts* (rho = 0.24, *p* < 0.01), *calls count* (rho = 0.23, *p* < 0.05)*, outgoing calls* (rho = 0.20, *p* < 0.05)*, and incoming calls* (rho = 0.28, *p* < 0.01) were significantly linked to age.

Next, gender differences were tested by means of a Mann–Whitney *U* test. With respect to the smartphone variables, males demonstrated higher values in *active contacts* (*Z* = -2.744, *p* < 0.01), *calls count* (*Z* = -3.317, *p* < 0.01), *incoming calls* (-3.782, *p* < 0.01), *outgoing calls* (*Z* = -2.977, *p* < 0.01) and *call duration in minutes* (*Z* = -2.221, *p* < 0.05).

**FIGURE 1:**
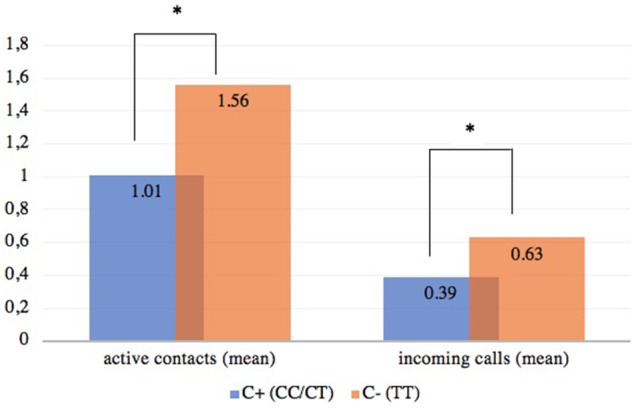
Association between the rs2268498 genotypes and the smartphone variables *active contacts* and *incoming calls. N*(C+) = 88, *n*(C–) = 29, ^∗^*p* < 0.05. Please note that the depicted significance is derived by the means of the Mann-Whitney *U* test. The means of the variables on the *Y* axis (and not the mean ranks) are presented here for reasons of clarity.

The results of a Mann–Whitney *U* test demonstrated that the TT-genotype (C- group) was linked to a significantly higher number of *active contacts* (*Z* = -2.313, *p* = 0.02) and significantly higher number of *incoming calls* (*Z* = -2.298, *p* = 0.02) (Figure 1). Due to the significant association between those variables and age, an ANCOVA with Blom-transformed variables was conducted where age was included as a covariate. The results with respect to the variable *active contacts* [*F*(1,114) = 3.890, *p* = 0.05] barely missed significance. The same was true for the variable *incoming calls* after age was controlled for [*F*(1,114) = 3.428, *p* = 0.07]. Moreover, no significant interactions between gender and rs2268498 on the smartphone variables *incoming calls* and *active contacts* could be observed, when gender was entered as a second independent variable. However, we point to the fact that searching for a gene by gender interaction is not meaningful in our study because the cell sizes were rather small (e.g., the number of male TT-carriers was 13).

Since in total eight smartphone variables were examined, we applied the Bonferroni method as a multiple-comparisons correction. The significance threshold was then *p* = 0.05/8 = 0.006 and none of the previously shown associations remained significant. However, please note that the Bonferroni correction is a very strict correction with the consequence of low power in statistical testing and a number of other disadvantages (we refer to Bender and Lange, 2001; the authors advise to use the Bonferroni correction when the number of tests is less than five). Additionally, since we set up a *directed* hypothesis on the relationship between the smartphone variables and the rs2268498, and did not test a random/large number of smartphone variables, we think that the results from the Bonferroni correction might be too strict and need to be interpreted with caution. In sum, we find it important to report the results of the present study (being in line with a large body of literature on this SNP), but again stress that the present findings should be understood as preliminary. From our perspective, the findings demonstrate the feasibility of linking molecular genetic markers with real-world variables and the here presented findings should be “only” seen as an illustration of this.

Extraversion (*M* = 4.37, *SD* = 0.91) was positively linked to all investigated smartphone variables. The correlations varied between rho = 0.20 and rho = 0.40 (*p* < 0.05). The rs2268498 genotypes and extraversion were not significantly linked.

## Discussion

The present work aimed to prove the feasibility to combine molecular genetic information in a meaningful way with real-world behavior, here size of the active social network tracked directly from the smartphone. We do not want to overstress the present results, because our sample is too small to claim general validity of our findings. Aside from this, the observed genetic association fits very well with the literature, again demonstrating that the TT-variant of rs2268498 is linked to higher prosocial behavior/prosocial abilities (Melchers et al., 2015, 2017), here in the light of a larger social network mirrored in the variable number of *active contacts*. Note that other genetic variations of the OXTR gene have been also investigated in the context of social neuroscience (Ebstein et al., 2012; Kumsta and Heinrichs, 2013), therefore other candidates on this gene clearly would have been interesting targets in the realm of the present work. Given that rs2268498 is one of the few, where functionality is likely/has been demonstrated (Reuter et al., 2017) and also in line with the rather straight forward findings so far (as cited), we focused on this single SNP.

Aside from the genetic link to this smartphone variable, the present study reveals several important notes for researchers interested in this new discipline coined *Psychoneuroinformatics* (Montag et al., 2016; see also Yarkoni, 2012; Markowetz et al., 2014 for an introduction into the term *Psychoinformatics*). Of note, other researchers speak in the realm of this new field of *digital phenotyping* (Onnela and Rauch, 2016; Insel, 2017), probably best achieved via methods of *Psychoinformatics*. First of all, an advantage of the present research approach to study the biological underpinnings of personality/sociality traits is the inclusion of information beyond self-report. E.g., if you ask a person how large his or her social network is, you might get biased data. Using smartphone variables such as the present ones gives you an exact estimate also with the advantage that one gets insights into the actual size of both the social network *per se*, but also the *active* social network. The importance to distinguish between these concepts (active vs. passive or complete social network) becomes visible, because in our work an association appeared only between rs2268498 and the active social network size. Using smartphone applications as the present one will also enable researchers to conduct more easily longitudinal research, also in the area of *Personality Neuroscience*.

Although real-world behavior is of great relevance to be included in future neuroscientific works (see evidence for the feasibility to combine MRI data with real-word data in Montag et al., 2017a), several problems arise. First, for the moment it is not that easy to recruit a large number of participants for biologically/neuroscientifically oriented works, investigating individual differences in human behavior, since applications have to be installed on smartphones or related devices together with gathering biomarkers. This naturally limits the inclusion of thousands of participants, as done in the impressive work by Luciano et al. (2018). In particular, in molecular genetics small sample sizes such as the present one represent a problem. In addition, researchers will need to find a standard on how often and how long variables from the Internet of Things need to be tracked to get stable insights into a person’s behavior. Or more generally spoken, the psychometric quality of predicting personality by real-world behavior tracked on smartphones need yet to be established. Additionally, more research is needed on the question if and how personality or the situational context might affect one’s smartphone use, and, in turn, affect the examined associations. First studies demonstrated divergent findings on this topic, with some studies reporting a positive link between smartphone use and social engagement (e.g., attending gatherings with friends and colleagues) (Kim et al., 2016), while others demonstrated using mobile smart devices less for online content or social activities when in social situations such as at a restaurant with friends or in an intimate moment with a partner (Vorderer et al., 2016). See also the new work by Dwyer et al. (2018) showing that smartphones reduce enjoyment of face-to-face interactions. Kushlev et al. (2019) even reported that smartphones reduce smiles between strangers. However, please note that several of the here mentioned studies used self-report data, where the answers might be biased through (a) information recall difficulties (e.g., frequency of smartphone use) or (b) social desirability (when participants need to report how often they use their phones in social situations, they might adapt their responses in accordance with social norms). Finally, problems regarding multiple testing arise. A trait such as extraversion impacts on many features of the smartphone. Therefore, it is very difficult to hypothesize on which exact variable on a smartphone the effect of a SNP, best linked to self-reported personality, can be observed. Please note that due to technical reasons (“sandbox principle”) it was not possible to take a look at activities inside social network applications such as WhatsApp. Moreover, tracking content in WhatsApp would raise further ethical concerns and might also lower recruitment success given the very intimate nature of one’s own content. However, since WhatsApp works with the telephone numbers, saved on our phones, we believe that the variable “contacts” examined in the present study also represents a reasonable approximate of a person’s WhatsApp contacts.

In sum, we stress at the end of this article again, that the presented findings should be understood as being illustrative of a new approach to do studies in the field of molecular genetic association studies.

## Data Availability Statement

Datasets are available on request: The raw data supporting the conclusions of this manuscript will be made available by the authors, without undue reservation, to any qualified researcher.

## Author Contributions

CM, RS, E-MR, and HB designed the present study. CM drafted the first version of the introduction and discussion, whereas RS drafted the first version of the method and result section. Both RS and E-MR were responsible for data-gathering and processing of smartphone variables. All authors worked over the manuscript again and approved its final draft.

## Conflict of Interest Statement

The authors declare that the research was conducted in the absence of any commercial or financial relationships that could be construed as a potential conflict of interest.

## Footnotes

1 https://www.ckannen.com

2 We computed the variable “active contacts” by running through/inspecting the call list of every participant per day. All numbers (from incoming, outgoing, and missed calls) were looked up in the contact list and saved to an active contact list. If there were duplicates, these were deleted. Also different phone numbers of one contact person were summed up to one active contact. At the end a mean over the 12 days of recordings was computed per participant.
